# Freezing of Gait in Parkinson's Disease Is Associated with Reduced 6-[^18^F]Fluoro-l-*m*-tyrosine Uptake in the Locus Coeruleus

**DOI:** 10.1155/2016/5430920

**Published:** 2016-02-23

**Authors:** Sayaka Asari Ono, Toshihiko Sato, Shin-ichi Muramatsu

**Affiliations:** ^1^Division of Neurology, Saitama Medical Center, Jichi Medical University, Saitama 330-8503, Japan; ^2^Utsunomiya Central Clinic, Tochigi 321-0112, Japan; ^3^Division of Neurology, Jichi Medical University, Tochigi 329-0498, Japan

## Abstract

Freezing of gait (FOG) is a common disorder in Parkinson's disease (PD) and could be attributed to a reduction in brain noradrenaline. The aim of this study was to determine the relationship between aromatic l-amino acid decarboxylase (AADC) activity in the locus coeruleus (LC) and FOG in PD using high-resolution positron emission tomography with an AADC tracer, 6-[^18^F]fluoro-l-*m*-tyrosine (FMT). We assessed 40 patients with PD and 11 age-matched healthy individuals. PD was diagnosed based on the UK Brain Bank criteria by two movement disorder experts. FOG was directly observed by the clinician and assessed using a patient questionnaire. FMT uptake in the LC, caudate, and putamen was analyzed using PMOD software on coregistered magnetic resonance images. FOG was present in 30 patients. The severity of FOG correlated with the decrease of FMT uptake in the LC regardless of disease duration and the severity of other motor impairments, indicating dysfunction of the noradrenergic network in FOG.

## 1. Introduction

Freezing of gait (FOG) is a debilitating and common symptom in Parkinson's disease (PD). More than 80% of patients with PD experience FOG during the course of the disease [1]. FOG is defined as “brief, episodic absence or marked reduction of forward progression of the feet despite the intention to walk [2, 3].” Patients feel as if their feet are “sticking or glued to the ground” for several seconds when initiating gait, turning, or walking through narrow spaces. FOG is frequently not responsive to dopamine replacement medication and is considered not just a pure motor phenomenon but probably caused by motor, affective, and cognitive deficits [4–6]. FOG may be attributed to the loss of noradrenergic neurons in the locus coeruleus (LC) and their projections to the frontal lobe [7]. Such an association was suggested by the observation that l-*threo*-3,4-dihydroxyphenylserine (l-DOPS or droxidopa), a precursor of noradrenaline, improves FOG in some patients with PD [8, 9]. To elucidate the relationship between FOG and catecholamine synthesis in the LC, we utilized positron emission tomography (PET) with 6-[^18^F]fluoro-l-*m*-tyrosine (FMT), a sensitive tracer for aromatic l-amino acid decarboxylase (AADC) [10, 11].

## 2. Methods

### 2.1. Subjects

Our sample consisted of 40 patients with PD and 11 age-matched healthy individuals. PD was diagnosed clinically according to the UK PD Society Brain Bank criteria [12, 13]. All of the patients had bradykinesia and at least one of the three features of PD: a 4–6 Hz resting tremor, rigidity, and postural instability. All of the patients had asymmetric onset and showed a positive response to dopaminergic medication. None exhibited atypical symptoms such as severe gaze palsy or symptomatic dysautonomia. The control group included healthy individuals with no history of neurologic or psychiatric diseases. FOG was defined as a score of one or more on item 3 of the Freezing of Gait Questionnaire (FOG-Q) [1], or if documented on examination by a movement disorder specialist. Motor symptoms were evaluated using the motor examination part of the Unified Parkinson's Disease Rating Scale (UPDRS). Global cognitive function was assessed with the Mini Mental State Examination (MMSE). Depression was assessed with the Geriatric Depression Scale (GDS). The institutional ethics committee approved this study, and all participants gave written informed consent.

### 2.2. PET Imaging

All patients stopped levodopa at least 16 h before their PET scan. All subjects took 2.5 mg/kg of oral carbidopa, a peripheral AADC inhibitor, 1 h before the FMT injection to increase the availability of the tracer. Prior to the emission scan, a 10 min transmission scan was obtained for attenuation correction. Subsequently, 0.12 mCi/kg of FMT in saline was infused into an antecubital vein, and a 30–90 min static three-dimensional acquisition scan was started simultaneously using a PET-CT scanner (GEMINI GXL, Philips, Amsterdam, Netherlands). Each subject also underwent 3.0-tesla MR imaging (Achieva 3.0 T, Philips) using an inversion recovery proton density-weighted pulse sequence to enhance the contrast of anatomical structures [11].

### 2.3. Volumes of Interest Analysis

Volumes of interest (VOIs) in the putamen, caudate nucleus, and LC were defined in three dimensions (3D) bilaterally on the coregistered MR images where these structures were best visualized. PMOD analysis software (PMOD Japan Inc., Tokyo) was used to manually delineate VOIs on MRIs to include the putamen, the head of caudate nucleus, and the LC. For reference, cerebellar 3D VOIs were also defined in the cerebellar cortex. Target region-to-cerebellum ratio (TCR) values of radioactivity were calculated in the 80–90 min frame for each structure, using bilaterally averaged cerebellar VOI data as the denominator. For analysis of their association with major motor symptoms in the PD subjects, TCR values of each structure were analyzed on the contralateral side from the more affected limbs.

### 2.4. Statistical Analysis

The degree of association between TCR values and scores of FOG-Q was analyzed by calculating Spearman's rank correlation coefficients. The partial correlation test was used to control for the effects of additional variables. Statistical tests were performed with SPSS (version 19, IBM), and the *α* level for significance was set at *P* < 0.05.

## 3. Results

### 3.1. Characteristics of Subjects

Demographic and clinical characteristics of the patients with PD and those of the control subjects are listed in Table 1. The mean ages of the patients with PD (13 male, 27 female) and the control subjects (5 male, 6 female) were 68.6 years (SD 8.5) and 64.5 years (SD 6.6), respectively. The patients exhibited a wide range of both duration and severity of symptoms. The mean duration of symptoms was 4.1 years (SD 4.0), and the mean UPDRS motor score was 27.2 (SD 14.0). The mean FOG-Q score was 8.6 (SD 6.2), the mean MMSE score was 25.2 (SD 4.3), and the mean GDS was 6.1 (SD 4.0).

### 3.2. Decrease of FMT Uptake in the LC

**Figure 1:**
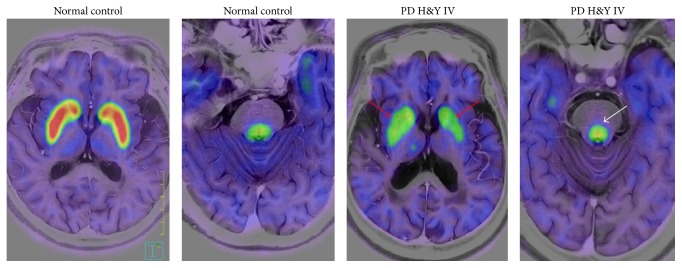
Representative FMT-positron emission tomography images of a healthy individual and a patient with advanced stage PD. Decrease of FMT uptake is obvious in the putamen and caudate (red arrow) in the PD patient. FMT uptake in the locus coeruleus (white arrow) in this patient is relatively maintained. H&Y: Hoehn and Yahr stage.

**Figure 2:**
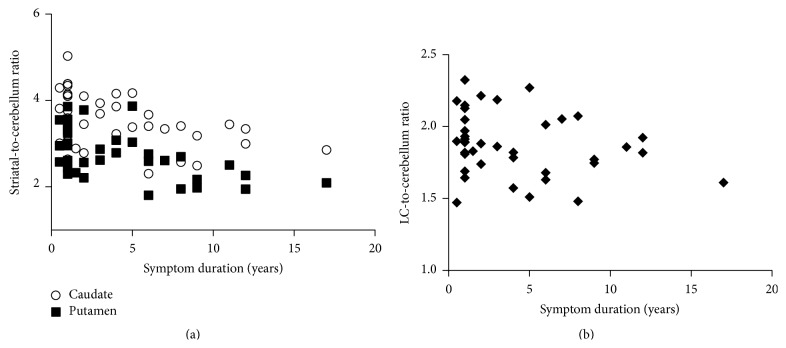
Scatter plots of FMT uptake against symptom duration in (a) the striatum contralateral to the more affected limb and (b) the locus coeruleus in patients with PD.

Figure 1 shows representative images of FMT uptake in a normal subject and in a patient with PD.  Figure 2(a) shows scatterplots of FMT uptake against disease duration in the caudate and the putamen, contralateral to the more affected limbs in the patients with PD. Similar plots are shown in the LC of PD patients (Figure 2(b)). Decline of FMT uptake—along with the disease duration—is more prominent in the striatum than in the LC.

### 3.3. Correlation of FOG and FMT Uptake

**Figure 3:**
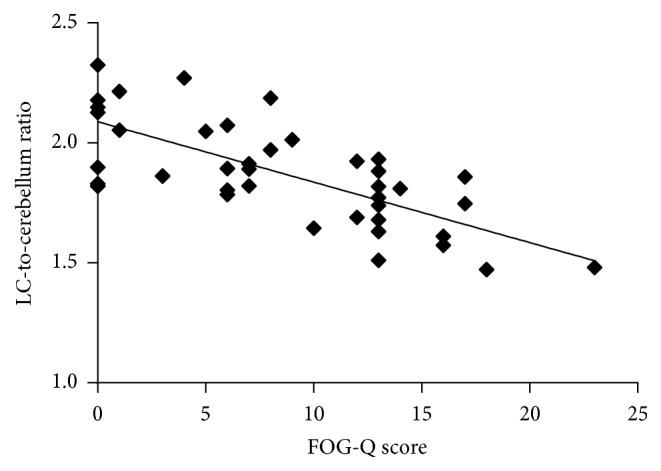
Scatter plots of FMT uptake against FOG-Q total score in the locus coeruleus of patients with PD.

When the correlation between FMT uptake and FOG severity was tested and the UPDRS III was entered into the analysis as a covariate, there were positive correlations between the severities of FOG and FMT uptake in the LC (*r* = −0.69, *P* < 0.01) (Figure 3). Neither MMSE nor GDS scores showed significant correlations with FMT uptake values in the LC.

## 4. Discussion

FOG is considered a distinct clinical motor feature of PD. FOG does not correlate with tremor, bradykinesia, or rigidity [3, 14]. Although FOG is more common in advanced stages of PD, it may occur early in the course of the disease and in untreated patients [3, 6]. The precise pathophysiological mechanism of FOG and the underlying neural network dysfunction are unknown. Clinical observations that FOG does not respond sufficiently to dopaminergic medication and that FOG is also observed during the “on” state suggest that nondopaminergic pathways are responsible for FOG [6, 7]. Previous studies using functional or structural imaging approaches yielded conflicting results and suggested that a variety of brain regions are linked to FOG, reflecting complex network dysfunction in FOG [15–17].

In this study, we focused our analysis on the LC. FMT is approximately twice as sensitive as 6-[^18^F]fluoro-l-dopa (FDOPA) as an AADC tracer. Higher spatial resolution of the FMT images enabled us to analyze AADC activity precisely in the LC [10, 11]. Postmortem investigations of patients with PD demonstrate a substantial loss of neurons in the LC, with a mean neuronal loss of 83% in later stages of the disease [18, 19]. Moreover, neurons in the LC are affected earlier than neurons in the substantia nigra pars compacta [20]. Consistent with these pathological observations, FMT uptake values in the LC decreased in the patients with PD regardless of disease duration. The LC plays a pivotal role in the noradrenergic networks in the brain. Neurons in the LC project their axons to widespread areas in the motor and sensory cortexes. Behavioral studies in rodents and nonhuman primates demonstrated that release of noradrenaline in response to a particular sensory event would provoke or facilitate dynamic reorganization of neural networks, creating a completely new functional network. This functional reconfiguration permits rapid behavioral adaptation to changing environmental imperatives [21]. Thus, dysfunction of the LC could be an underlying mechanism that causes patients with FOG to exhibit delayed motor switching [22, 23] and poor performance on tests that require higher attentional demands [24]. Although cognitive decline and depression can negatively influence gait and balance [25], we did not find significant associations either between FOG-Q and MMSE scores or between FOG-Q and GDS scores in our patients with PD.

## 5. Conclusions

In patients with PD with FOG, the severity of FOG was associated with the decrease of FMT uptake values in the LC. Dysfunction of noradrenergic networks in the brain seems to be the basis for FOG in PD.

## Figures and Tables

**Table 1:** Clinical characteristics of the subjects.

Characteristics	PD	Normal control
Age, years	68.6 ± 8.5	64.5 ± 6.6
Male/female	13/27	5/6
MMSE	25.2 ± 4.3	28.3 ± 1.6
Symptom duration, years	4.1 ± 4.0	
UPDRS part III motor score	27.2 ± 14	
FOG-Q total score	8.6 ± 6.2	

FOG-Q: Freezing of Gait Questionnaire; MMSE: Mini Mental State Examination; UPDRS: Unified Parkinson's Disease Rating Scale. Data are given as mean ± standard deviation values.
